# Morbidity and Long-Term Mortality Predictors Following Isolated Mitral Valve Replacement: A Single-Center Cohort Study on the Effect of Sex

**DOI:** 10.3390/jcdd13060252

**Published:** 2026-06-07

**Authors:** Rauf Önder, Salih Özçobanoğlu

**Affiliations:** 1 Department of Cardiovascular Surgery, Gaziantep City Hospital, Gaziantep 27470, Turkey; 2 Department of Cardiovascular Surgery, Akdeniz University Faculty of Medicine, Antalya 07070, Turkey; sozcobanoglu@akdeniz.edu.tr

**Keywords:** mitral valve replacement, sex, postoperative morbidity, long-term mortality, survival

## Abstract

**Objective:** The present study aimed to determine clinical and surgical variables associated with postoperative morbidity and 10-year mortality in isolated mitral valve replacement (MVR) and to assess the association between sex and postoperative outcomes. **Materials and Methods:** A total of 1629 patients undergoing isolated MVR in one center during the period between January 2000 and December 2015 were retrospectively analyzed. Hospital records provided demographic, clinical, echocardiographic, and operative data. Cox regression analyses were used to determine factors associated with postoperative morbidity and long-term mortality. The Kaplan–Meier method was used to analyze long-term survival, and the log-rank test was used to compare the groups. **Results:** A total of 866 (53.1%) patients were male and 763 (46.9%) were female, and the average age was 63.8 ± 10.9 years. There were no significant differences in female and male patients regarding basic demographic and clinical characteristics. The first 30-day in-hospital morbidity rate was also significantly greater in women than in men (25.7% vs. 20.6%; *p* = 0.015). The in-hospital mortality was more prevalent among women (5.0% vs. 3.0%; *p* = 0.043). Age, sex (female), diabetes mellitus, pulmonary hypertension, chronic obstructive pulmonary disease, critical preoperative condition, high body mass index, longer cardiopulmonary bypass time, and low left ventricular functioning were significantly associated with postoperative morbidity in multivariable analysis. The total mortality rate during a 10-year follow-up was 33.2%, which was considerably higher among women compared to men (36.3 vs. 30.5; *p* = 0.013). Kaplan–Meier analysis demonstrated significantly lower long-term survival in female patients (log-rank *p* = 0.011). **Conclusions:** Morbidity and mortality following isolated MVR are closely related to patient-related factors. Female sex showed a significant adjusted association with higher 10-year mortality in multivariable analysis, warranting careful long-term risk assessment in female patients.

## 1. Introduction

Mitral valve pathologies are one of the most frequently treated valve diseases in cardiac surgery [1,2]. Although mitral valve repair is generally performed in these diseases, mitral valve replacement (MVR) is preferred as the primary treatment when the anatomy is unsuitable [1,2]. Replacement surgery can also be performed for reasons such as infective endocarditis, severe annular calcification, or severe dystrophic changes in valvular tissue that threaten valve durability [1,2,3]. However, it has been reported in the literature that MVR is a high-risk surgery in the perioperative period, with an in-hospital mortality rate ranging from 4% to 7% [1,4,5].

The complex valve anatomy and advanced pathological findings, which are more frequently encountered in female patients, increase the likelihood of choosing replacement procedures rather than valve repair [6,7]. However, the effect of sex on the outcomes of MVR surgery is still a controversial topic in the literature [4,8]. Analyses have shown that mortality rates are higher in women than in men, while other studies indicate that sex does not affect short- or long-term survival [4,8,9,10]. On the other hand, many parameters other than sex have been identified as affecting MVR mortality and morbidity. Factors such as advanced age, low ejection fraction (EF), anemia, and prolonged cardiopulmonary bypass (CPB) duration are associated with mortality [9]. In addition, concomitant diseases such as obesity, diabetes mellitus (DM), chronic obstructive pulmonary disease (COPD), and pulmonary hypertension (PH) have been reported to increase the risk of morbidity after mitral valve surgery [9,11,12]. Unlike previous studies focusing mainly on short-term postoperative outcomes, the present study evaluates both early postoperative morbidity and 10-year long-term mortality in a large cohort of patients undergoing isolated mitral valve replacement. Furthermore, by analyzing a homogeneous population undergoing isolated MVR without concomitant procedures, this study aims to clarify whether female sex shows a significant adjusted association with adverse outcomes beyond traditional clinical risk factors.

In this context, our study aimed to examine patients who underwent isolated MVR using one of the largest patient-cohort series in the literature and to better clarify the effects of clinical and surgical parameters, along with sex, on postoperative morbidity and long-term mortality.

## 2. Materials and Methods

### 2.1. Study Design and Ethical Approval

This study is a retrospective cohort study including patients who underwent mitral valve replacement surgery between January 2000 and December 2015. Prior to the study, approval was obtained from the Clinical Research Ethics Committee of Akdeniz University Faculty of Medicine (Decision No.: KAEK-920, Date: 13 December 2023). The research was conducted in accordance with the principles of the Helsinki Declaration. Due to the retrospective study design, informed consent was not obtained from the patients.

### 2.2. Inclusion and Exclusion Criteria

**Figure 1:**
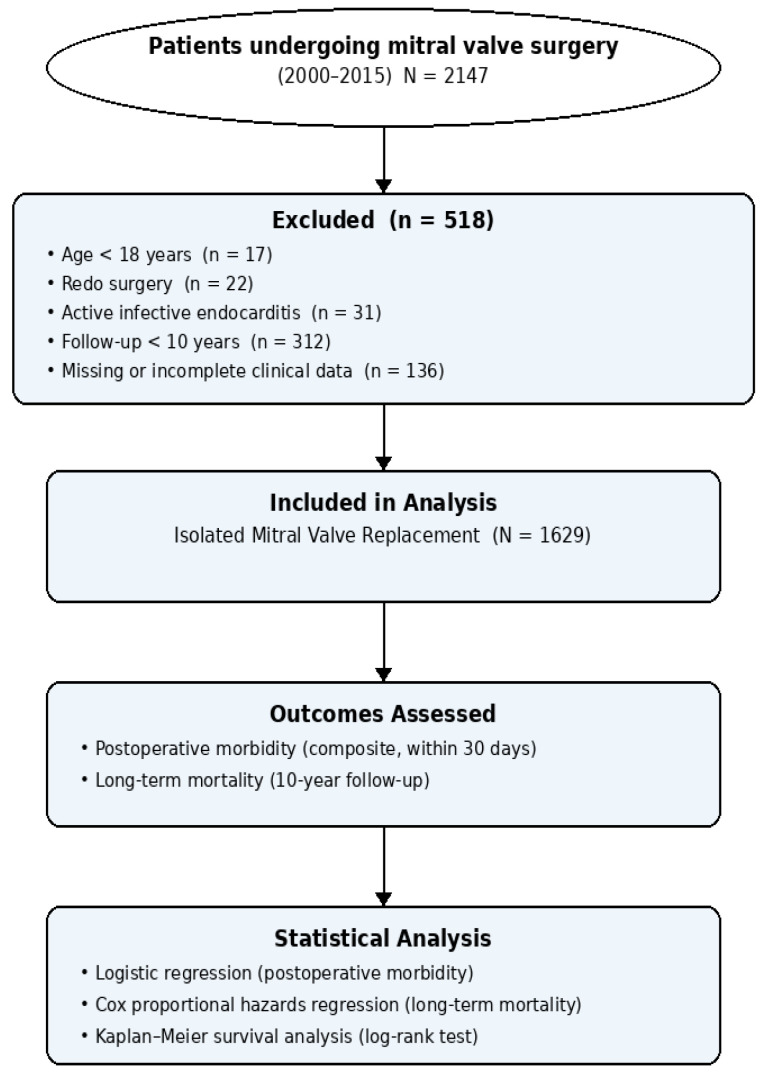
Flow diagram of patient selection for the study cohort undergoing isolated mitral valve replacement.

A total of 2147 patients who had previously undergone mitral valve surgery were included in the evaluation. In order to ensure that the study was conducted in a homogeneous patient population, patients under 18 years of age, patients undergoing redo surgery, patients diagnosed with active infective endocarditis, and patients with a follow-up period of less than 10 years or lacking basic clinical data were excluded from the study. Of the 518 excluded patients, 312 were excluded due to a follow-up period shorter than 10 years and 136 due to missing or incomplete baseline clinical data. After applying these exclusion criteria, 1629 patients who underwent isolated mitral valve replacement (MVR) formed the final analysis group. The prosthetic valves used in the study included the Medtronic Hall mechanical valve (Medtronic Inc., Minneapolis, MN, USA) among the mechanical prostheses. The patient selection and exclusion process is summarized in the study flow diagram in Figure 1.

### 2.3. Data Collection and Variable Identification

Demographic, clinical, and operative data of the patients were retrospectively obtained from hospital information systems. Demographic and clinical data included age, sex, body mass index (BMI), hypertension (HT), diabetes mellitus (DM), chronic obstructive pulmonary disease (COPD), pulmonary hypertension (PH), and coronary artery disease (CAD). Preoperative evaluation considered the presence of atrial fibrillation (AF), critical preoperative status (CPC), serum creatinine levels, and echocardiographically determined left ventricular (LV) function. Operative variables included cardiopulmonary bypass (CPB) duration, aortic cross-clamp (ACC) duration, and valve type used (mechanical or bioprosthetic). The prosthetic valves used in the study included the Medtronic Hall mechanical valve (Medtronic Inc., Minneapolis, MN, USA) among the mechanical prostheses. Prosthesis type was recorded and included in the baseline description (Table 1); however, it was not retained in the final multivariable models because it was not associated with sex distribution or the study outcomes in preliminary analyses. Nevertheless, residual confounding related to prosthesis characteristics cannot be fully excluded. Left ventricular function was categorized based on left ventricular ejection fraction (LVEF) according to preoperative transthoracic echocardiography findings. Accordingly, patients with LVEF ≥ 50% were classified as having good left ventricular function, those with LVEF between 35–49% as having moderate left ventricular function, and those with LVEF < 35% as having poor left ventricular function. Experienced cardiologists performed all echocardiographic evaluations according to standard measurement protocols. Critical preoperative status was defined as the patient being considered hemodynamically or metabolically unstable in the preoperative period. Postoperative atrial fibrillation was defined as new-onset atrial fibrillation occurring within 30 days after surgery in patients without preoperative AF.

### 2.4. Defining Endpoints

Two key endpoints were identified in the study: postoperative morbidity and long-term mortality. Postoperative morbidity included complications such as cerebrovascular events (CVE), re-operation due to bleeding, pulmonary failure or the need for prolonged mechanical ventilation, sepsis, acute kidney injury (AKI), and deep sternal surgical site infection. A composite morbidity endpoint (defined as the occurrence of at least one of these complications within 30 days) was used to maximize statistical power for sex-based comparisons and to align with prior literature on cardiac surgical outcomes; individual complication rates are reported separately in Table 2 to allow granular interpretation. Long-term mortality was assessed as 10-year all-cause mortality among patients with complete 10-year follow-up. Each patient was followed for exactly 10 years from the date of their index operation; patients operated on between January 2000 and December 2015 therefore had follow-up extending until January 2010 and December 2025, respectively. Mortality data were validated by integrating hospital records with the national mortality registry, with the final data extraction completed in December 2025. Patients with follow-up shorter than 10 years were excluded rather than treated as censored observations; this was acknowledged as a methodological limitation. Acute kidney injury (AKI) was defined according to the Kidney Disease: Improving Global Outcomes (KDIGO) criteria as an increase in serum creatinine ≥ 0.3 mg/dL within 48 h or ≥1.5 times baseline within 7 days. Prolonged mechanical ventilation was defined as the requirement for mechanical ventilation for more than 24 h after surgery.

### 2.5. Sample Size and Power Assessment

Since this study had a retrospective cohort design, the sample size was not predetermined; all eligible patients who underwent isolated mitral valve replacement (MVR) during the defined time period and met the inclusion criteria were included in the analysis. Ultimately, the study included 1629 patients, with sufficient statistical power to assess clinical and surgical predictors of postoperative morbidity and long-term mortality. With the current sample size, it was calculated that an absolute difference in mortality rate of ≥2% between female and male patients could be detected at a two-sided significance level of 5% and with statistical power above 80%.

### 2.6. Statistical Analysis

The statistical data were analyzed using R software (version 4.3.2; R Foundation for Statistical Computing, Vienna, Austria). Continuous variables were described as being in the form of mean and standard deviation, as well as in the form of numbers and percentages in categorical variables. The predictors of postoperative morbidity and long-term mortality were determined through Cox proportional hazards regression and logistic regression; variables with *p*-values < 0.20 in the univariate analysis were included in the multivariate model. To account for the competing risk of in-hospital mortality, a Fine–Gray subdistribution hazard model was additionally performed for the long-term mortality endpoint. The proportional hazards assumption for the Cox model was verified using Schoenfeld residuals; no significant violation was detected (global test *p* > 0.05). The goodness-of-fit of the logistic regression model was assessed using the Hosmer–Lemeshow test (χ^2^ = 1.678, df = 4, *p* = 0.795), indicating adequate model calibration. The Kaplan–Meier method was used for preliminary survival visualization; final long-term survival curves were generated from the Cox proportional hazards regression model, adjusted for all covariates included in the multivariable analysis, and differences between groups were assessed using the log-rank test; early postoperative mortality (within 30 days) was analyzed separately as a distinct endpoint. As detailed in the Defining Endpoints section, all patients completed the full 10-year follow-up period; accordingly, no censored observations were present in the survival analysis and censoring marks are not displayed in the survival figure. A two-sided *p*-value of less than 0.05 is considered significant in all statistical calculations.

## 3. Results

A total of 1629 patients who underwent isolated mitral valve replacement were included in the study; 866 (53.1%) were male, and 763 (46.9%) were female. The mean age was 63.8 ± 10.9 years, and no significant difference was found between male and female patients (*p* = 0.146). There were no statistically significant differences between sex in body mass index, hypertension, diabetes mellitus, pulmonary hypertension, chronic obstructive pulmonary disease, coronary artery disease, distribution of left ventricular function, history of previous percutaneous coronary intervention, critical preoperative status, and serum creatinine levels above 200 µmol/L. Preoperative atrial fibrillation was present in 598 patients (36.7%), with the rate being 38.2% in men and 35.0% in women, showing no significant difference between the groups (*p* = 0.182). Baseline demographic and preoperative clinical characteristics according to sex are summarized in Table 1.

Cardiopulmonary bypass time (121.6 ± 27.8 min) and aortic cross-clamp time (85.5 ± 22.3 min) were similar between female and male patients (*p* = 0.581 and *p* = 0.176, respectively); the ratio of mechanical to bioprosthetic valves used did not differ between the groups (*p* = 0.743). In the early postoperative period, atrial fibrillation was observed in 267 patients (16.4%), acute renal injury in 167 patients (10.3%), and cerebrovascular events in 62 patients (3.8%), and no significant difference was found between sex in terms of these complications (*p* = 0.121, *p* = 0.364, and *p* = 0.613, respectively). The need for re-operation due to bleeding was significantly higher in female patients compared to male patients (4.8% vs. 2.3%; *p* = 0.005); prolonged mechanical ventilation was more frequent in women, although this difference was not statistically significant (12.1% vs. 9.1%; *p* = 0.054); and there was no significant difference between the groups in terms of sepsis (2.7%) and deep sternal surgical site infection (1.5%). Composite in-hospital morbidity, defined as the development of at least one postoperative complication within the first 30 days, was detected in a total of 374 patients (22.9%) and was significantly higher in female patients compared to male patients (25.7% vs. 20.6%; *p* = 0.015). The in-hospital mortality rate was 3.9% overall and was significantly higher in women than in men (5.0% vs. 3.0%; *p* = 0.043) (Table 2).

In logistic regression analysis, advanced age (OR = 1.02; 95% CI: 1.01–1.04; *p* < 0.001), female sex (OR = 1.22; 95% CI: 1.03–1.45; *p* = 0.022), diabetes mellitus (OR = 1.39; 95% CI: 1.08–1.78; *p* = 0.009), pulmonary hypertension (OR = 1.42; 95% CI: 1.01–2.01; *p* = 0.045), chronic obstructive pulmonary disease (OR = 1.53; 95% CI: 1.12–2.10; *p* = 0.008), critical preoperative status (OR = 1.86; 95% CI: 1.24–2.78; *p* = 0.003), increased BMI (OR = 1.03; 95% CI: 1.00–1.06; *p* = 0.048), prolonged cross-clamp time (OR = 1.02; 95% CI: 1.01–1.04; *p* = 0.039), and poor left ventricular function (OR = 1.41; 95% CI: 1.00–1.98; *p* = 0.049) were identified as significant multivariable predictors of postoperative morbidity (Table 3). The Hosmer–Lemeshow goodness-of-fit test confirmed adequate model calibration (χ^2^ = 1.678, df = 4, *p* = 0.795).

In long-term mortality analysis, advanced age, female sex, pulmonary hypertension, critical preoperative condition, serum creatinine level above 200 µmol/L, increased body mass index, and poor left ventricular function were found to be associated with mortality, while in multivariate analysis, advanced age (HR = 1.04; 95% CI: 1.02–1.06; *p* < 0.001), female sex (HR = 1.24; 95% CI: 1.06–1.55; *p* = 0.013), pulmonary hypertension (HR = 1.43; 95% CI: 1.01–2.04; *p* = 0.045), critical preoperative condition (HR = 1.74; 95% CI: 1.08–2.81; *p* = 0.022), and serum creatinine level > 200 µmol/L were associated with mortality (Table 4). The proportional hazards assumption of the Cox model was verified using scaled Schoenfeld residuals; no significant violation was detected for any covariate (global test *p* > 0.05), confirming the validity of the model. To account for the competing risk of in-hospital mortality—which differed significantly between sexes (5.0% vs. 3.0%; *p* = 0.043)—a Fine–Gray subdistribution hazard model was additionally performed. The subdistribution hazard ratio (sHR) for female sex was 1.23 (95% CI: 1.05–1.68; *p* = 0.018), consistent with the Cox model estimate, confirming that the association between female sex and long-term mortality remains significant after competing risk adjustment.

**Figure 2:**
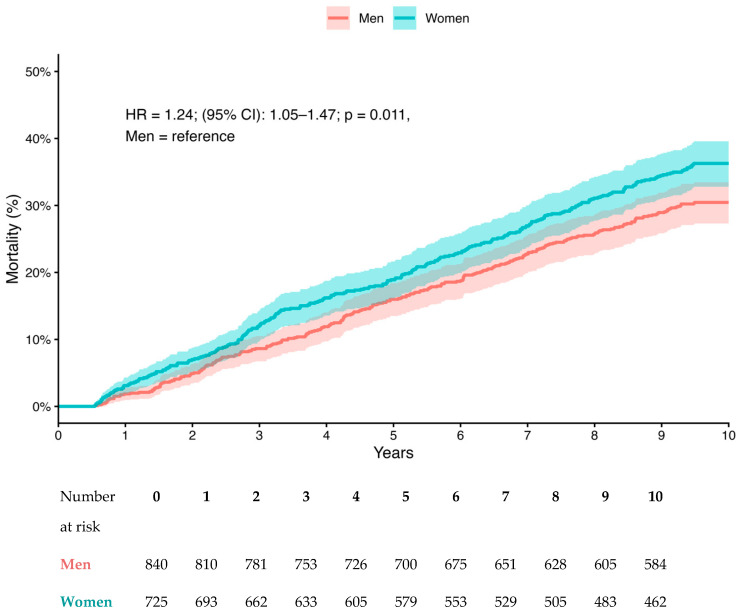
Long-term survival curves according to sex after isolated mitral valve replacement. Cox regression-based survival curves comparing long-term survival between female and male patients after isolated mitral valve replacement, adjusted for all covariates included in the multivariable model. This represents a conditional survival analysis conducted among patients who survived beyond 30 days postoperatively; early postoperative mortality (within 30 days) was analyzed separately and is not included in this survival analysis. The shaded areas represent the 95% confidence intervals around each survival estimate. Numbers at risk at each time point are provided below the figure.

The cumulative mortality rate over the 10-year follow-up period was 33.2% overall. The ten-year mortality rate was significantly higher in female patients compared to male patients (36.3% vs. 30.5%; *p* = 0.013). Kaplan–Meier survival analysis also showed that long-term survival was significantly lower in female patients compared to male patients (log-rank *p* = 0.011) (Figure 2).

## 4. Discussion

In mitral valve surgery, age and accompanying comorbidities are known to determine postoperative clinical outcomes and survival [10] significantly. However, studies on sex report higher mortality and morbidity rates in women; the fact that women also have poorer prognostic features in studies makes the sex factor questionable as an independent factor [4]. In light of these data, the main finding of our study is that female sex showed a significant adjusted association with higher morbidity and long-term mortality in multivariable analyses, after accounting for available clinical covariates. In addition, age, critical preoperative status, creatinine level, poor LV function, pulmonary hypertension, and BMI were identified as other significant predictors of long-term mortality. It should be noted that the predominant mitral valve pathology in this cohort was rheumatic in etiology, consistent with the epidemiological profile of cardiovascular disease in Turkey; this may limit the generalizability of findings to populations where degenerative etiologies predominate.

The observed higher morbidity among female patients is likely multifactorial rather than attributable to sex alone. Several potential mechanisms may explain this association. Women undergoing mitral valve surgery often have smaller body surface area and valvular and annular dimensions, which may increase procedural complexity. In addition, women may be referred for surgery at a later stage of the disease, potentially with more advanced pulmonary hypertension, myocardial remodeling, or symptomatic burden. Differences in comorbidity patterns, frailty, and unmeasured clinical severity markers may also contribute to these findings. Therefore, the association observed in the present study should be interpreted as reflecting a more complex risk profile rather than a direct biological effect of sex alone.

It has been reported that female patients are at higher risk for cardiac surgery because they often present with more advanced disease [4,13,14,15]. Furthermore, higher pulmonary artery pressure has been found in some cohorts in women, suggesting it is a significant factor contributing to increased surgical risk [4]. On the other hand, additional risk factors such as severe tricuspid regurgitation and advanced heart failure symptoms (NYHA Class IV) during surgery have been reported to be much higher in women than in men [16]. In addition, it has been shown that female patients undergoing MVR have surgery at an older age (61 vs. 64 years) [4]. Therefore, preoperative risk scores (EuroSCORE I) are significantly higher in female patients than in men (9.5% vs. 7.2%) [4]. Furthermore, studies have suggested that the mortality risk in women aged 40–59 (perimenopausal) is approximately 2.5 times higher than in men with similar risk factors, and that this is due to hormonal changes [8]. In our study, female sex was significantly associated with long-term mortality after MVR in multivariable analysis (HR = 1.24, *p* = 0.013). It should be noted that certain clinically relevant variables—including mitral valve etiology, NYHA functional class, right ventricular function, and prosthesis-related characteristics—were not available for inclusion in the model; therefore, this finding is best interpreted as a significant adjusted association rather than definitive evidence of an independent causal effect of sex. These results suggest that surgical risk may be influenced by sex-specific factors affecting long-term prognosis, and prospective studies with more comprehensive covariate adjustment are needed to further elucidate this relationship. One of the critical variables affecting clinical outcomes and mortality after MVR is patient age. In cases with postoperative mortality, a higher average age (approximately 62 vs. 43.4 years) was observed compared to the survival group [4,9]. In particular, it was found that the one-year mortality risk after MVR can reach 27.7% in the ≥75-year age group, with the effect of accompanying comorbidities. In contrast, mortality is below 1% in cases <65 years [10,16]. In our study, consistent with the literature, advanced age was a significant risk factor for isolated post-MVR morbidity (OR = 1.02, *p* < 0.001) and long-term mortality (HR = 1.04, *p* < 0.001).

It has been reported that 9.4% to 31.4% of patients undergoing MVR have a diagnosis of DM. In extensive patient series analyses, DM is an independent risk factor for in-hospital mortality and long-term survival (HR: 1.604) [17,18]. However, studies also report that it increases rehospitalization, especially in the first year after MVR, but does not increase mortality [10]. Although DM was found to be an independent and significant risk factor for morbidity in our study (OR = 1.39, *p* = 0.009), no independent association with long-term mortality was observed. The differing findings in the literature may be due to the need for more selective surgery in patients with diabetes for risk management, heterogeneous patient groups in the studies, and limited sample sizes. High BMI has also been identified as a significant risk factor for mitral valve surgery [19]. High BMI is strongly associated with PH and prolonged CPB time [19,20]. Obesity has been shown to increase the incidence of AKI in the postoperative period (17% vs. 5.2%), and the risk of AKI is much more pronounced (42%) in the subgroup of obese patients with low EF (<50%) [19]. In our study, increased BMI was a significant risk factor for both postoperative morbidity (OR = 1.03, *p* = 0.048) and long-term mortality (HR = 1.04, *p* = 0.014) and could affect the clinical course independently of comorbidities.

It has been determined that one of the predictors of short- and long-term mortality after MVR is pulmonary hypertension (PH), and that every 10 mmHg increase in mean preoperative pulmonary artery systolic pressure (PASP) increases mortality by 1.38 times [21]. Different cut-off values have been reported for target PASP before MVR, and studies in the literature recommend performing surgery without exceeding thresholds of <50 mmHg or <40 mmHg [20,22,23]. In addition, PH is an important risk factor for intraoperative mortality. While the intraoperative mortality rate is 2% in patients without PH, it has been shown to reach up to 12% in severe PH cases [20]. On the other hand, although PASP shows some regression in the early period after MVR, it has been found that in approximately 35% of patients with a preoperative diagnosis of PH, PASP remains high even two years after the operation, and persistent PH (PASP ≥ 35 mmHg) increases the risk of mortality [21]. Our study also showed that PH is a significant risk factor for postoperative morbidity (OR = 1.42, *p* = 0.045) and long-term mortality (HR = 1.43, *p* = 0.045).

COPD is also known to be a significant risk factor for mortality and first-year rehospitalization [10]. Although it is not a definitive risk factor for short-term mortality, studies have shown that it negatively affects long-term survival (HR: 1.75) [17]. It has also been reported to lead to prolonged mechanical ventilation needs and longer intensive care unit stays in the postoperative period [17]. In our study, although COPD was shown to be a risk factor for morbidity (OR = 1.53, *p* = 0.008), no association was shown with long-term mortality. The different findings in the literature suggest that differences in the definition of COPD and disease severity across studies, as well as the heterogeneity of the surgeries performed, may be the reason. It is known that 6% of patients undergoing MVR have a severe clinical condition when they have surgery [4]. CPC has been found to increase the risk of in-hospital mortality and to lead to approximately 11 times higher risk of death (univariate HR: 11.30). LV function also determines short- and long-term survival after mitral valve surgery [9]. In those with EF < 50%, the risk of in-hospital mortality is increased by 6.4 times, and it plays a direct determining role in one-year and long-term mortality (HR: 1.43) and the need for redo [9]. Similarly, in our study, it was found to be an independent risk factor for postoperative morbidity (OR = 1.86, *p* = 0.003) and long-term mortality (HR = 1.74, *p* = 0.022). It has been shown that poor LV function significantly increases both morbidity (OR = 1.41, *p* = 0.049) and long-term mortality (HR = 1.87, *p* = 0.002).

### Limitations

Firstly, we believe the study’s retrospective, single-center design is its primary limitation. The inability to include results by prosthesis type, surgical technique, and perioperative treatment strategy, as well as the inability to differentiate mortality causes into cardiac and non-cardiac, can also be considered limitations. Furthermore, patients with follow-up periods shorter than 10 years were excluded rather than included as censored observations, which may introduce selection bias; this approach was chosen to ensure a homogeneous cohort but limits the full use of available survival data. Important covariates such as mitral valve etiology, NYHA functional class, right ventricular function, and prosthesis-related characteristics were not available for inclusion in the multivariable models, which means that the association between female sex and adverse outcomes should be interpreted as an adjusted association rather than a definitive independent effect. Additionally, the study period spans 2000 to 2015, during which changes in operative technique, myocardial protection strategies, perioperative care, and prosthesis selection may have occurred; although the single-center design and standardized institutional protocols partially mitigate this era effect, it cannot be fully excluded as a confounding factor. Hospital readmission data were not systematically captured in this dataset and therefore could not be included as an outcome measure. Despite these limitations, the analysis of a large cohort of patients undergoing isolated MVR in a homogeneous patient population is a key strength of the study.

## 5. Conclusions

Our study evaluated in detail the effect of sex on early and late postoperative outcomes in isolated MVR cases. It was found that morbidity was influenced by patient clinical presentation and surgical process factors, and female sex showed a significant adjusted association with both morbidity and long-term mortality outcomes in multivariable analyses. While these findings highlight the importance of sex-specific risk assessment in MVR, they should be interpreted cautiously given the unavailability of certain covariates such as mitral valve etiology and NYHA class. Therefore, when conducting risk assessments for MVR cases, in addition to focusing on perioperative complications, risk factors that may affect long-term prognosis, especially in female patients, should be considered more carefully. Prospective, multicenter studies in this area will help identify the underlying mechanisms of sex-specific differences and develop long-term postoperative follow-up strategies.

## Figures and Tables

**Table 1:** Baseline demographic and preoperative clinical characteristics of patients undergoing isolated mitral valve replacement according to sex.

Variable	Total (*n* = 1629)	Male (*n* = 866)	Female (*n* = 763)	*p* Value
Age, years (mean ± SD)	63.8 ± 10.9	63.4 ± 11.7	64.2 ± 9.9	0.146
BMI, kg/m^2^ (mean ± SD)	26.6 ± 4.4	26.9 ± 3.9	26.3 ± 5.1	0.061
Presence of HT, *n* (%)	849 (52.1)	447 (51.6)	402 (52.7)	0.666
Presence of DM, *n* (%)	311 (19.1)	163 (18.8)	148 (19.4)	0.751
Presence of PH, *n* (%)	108 (6.6)	56 (6.5)	52 (6.8)	0.822
Presence of COPD, *n* (%)	239 (14.6)	129 (14.9)	110 (14.4)	0.774
Presence of CAD, *n* (%)	456 (27.9)	245 (28.3)	211 (27.6)	0.742
LV function, *n* (%)				0.684
Good	879 (54.0)	470 (54.3)	409 (53.6)	
Moderate	524 (32.1)	275 (31.8)	249 (32.6)	
Poor	226 (13.9)	121 (14.0)	105 (13.8)	
Prior PCI, *n* (%)	122 (7.5)	67 (7.7)	55 (7.2)	0.691
Preoperative AF, *n* (%)	598 (36.7)	331 (38.2)	267 (35.0)	0.182
CPC, *n* (%)	88 (5.4)	45 (5.2)	43 (5.6)	0.712
Serum creatinine > 200 µmol/L, *n* (%)	95 (5.8)	52 (6.0)	43 (5.6)	0.743

Abbreviations: SD, standard deviation; BMI, body mass index; HT, hypertension; DM, diabetes mellitus; PH, pulmonary hypertension; COPD, chronic obstructive pulmonary disease; CAD, coronary artery disease; PCI, percutaneous coronary intervention; AF, atrial fibrillation; CPC, critical preoperative condition; LV, left ventricular.

**Table 2:** Sex-based comparison of procedural characteristics and early postoperative outcomes after isolated mitral valve replacement.

Variable	Total (*n* = 1629)	Male (*n* = 866)	Female (*n* = 763)	*p* Value
CPB time, min (mean ± SD)	121.6 ± 27.8	121.2 ± 30.2	122.1 ± 25.4	0.581
ACC time, min	85.5 ± 22.3	85.1 ± 24.1	86.6 ± 19.2	0.176
Valve type, *n* (%)				0.738
Mechanical	1298 (79.3)	693 (80.0)	605 (79.3)	
Bioprosthetic	331 (20.7)	173 (20.0)	158 (20.7)	
Postoperative AF, *n* (%)	267 (16.4)	130 (15.0)	137 (18.0)	0.121
AKI, *n* (%)	167 (10.3)	83 (9.6)	84 (11.0)	0.364
Postoperative CVE, *n* (%)	62 (3.8)	31 (3.6)	31 (4.1)	0.613
Reoperation for bleeding, *n* (%)	57 (3.5)	20 (2.3)	37 (4.8)	0.005
Prolonged mechanical ventilation, *n* (%)	171 (10.5)	79 (9.1)	92 (12.1)	0.054
Sepsis, *n* (%)	44 (2.7)	19 (2.2)	25 (3.3)	0.179
Deep sternal surgical site infection, *n* (%)	24 (1.5)	11 (1.3)	13 (1.7)	0.469
Composite in-hospital morbidity (≥1 complication), *n* (%)	374 (22.9)	178 (20.6)	196 (25.7)	0.015
In-hospital mortality, *n* (%)	64 (3.9)	26 (3.0)	38 (5.0)	0.043

Abbreviations: CPB, cardiopulmonary bypass; ACC, aortic cross-clamp; SD, standard deviation; CVE, cerebrovascular event; AKI, acute kidney injury; AF, atrial fibrillation. Early postoperative outcomes were defined as events occurring within 30 days after surgery. Composite morbidity was defined as the occurrence of at least one postoperative complication.

**Table 3:** Predictors of postoperative morbidity after isolated mitral valve replacement: univariate and multivariate logistic regression analysis.

Variable	Univariate OR (95% CI)	*p*	Multivariate OR (95% CI)	*p*
Age (per year)	1.03 (1.02–1.05)	<0.001	1.02 (1.01–1.04)	<0.001
Female sex	1.33 (1.06–1.67)	0.015	1.22 (1.03–1.45)	0.022
Presence of HT	1.12 (0.93–1.36)	0.231	—	—
Presence of DM	1.46 (1.15–1.85)	0.002	1.39 (1.08–1.78)	0.009
Presence of PH	1.58 (1.11–2.25)	0.011	1.42 (1.01–2.01)	0.045
Presence of COPD	1.71 (1.28–2.28)	<0.001	1.53 (1.12–2.10)	0.008
Presence of CPC	2.08 (1.43–3.04)	<0.001	1.86 (1.24–2.78)	0.003
Serum creatinine > 200 µmol/L	1.39 (1.02–1.90)	0.037	1.27 (0.92–1.76)	0.145
BMI (per kg/m^2^)	1.04 (1.01–1.07)	0.012	1.03 (1.00–1.06)	0.048
CPB time (per min)	1.01 (1.00–1.02)	0.058		
ACC time (per min)	1.05 (1.02–1.08)	0.007	1.02 (1.01–1.04)	0.039
LV function-Good	Reference			
Moderate	1.14 (0.90–1.44)	0.282	—	—
Poor	1.69 (1.22–2.34)	0.002	1.41 (1.00–1.98)	0.049

Variables with *p* < 0.20 in univariate analysis were included in the multivariate model. Abbreviations: OR, odds ratio; CI, confidence interval; BMI, body mass index; CPB, cardiopulmonary bypass; ACC, aortic cross-clamp; HT, hypertension; DM, diabetes mellitus; PH, pulmonary hypertension; CPC, critical preoperative condition; LV, left ventricular; COPD, chronic obstructive pulmonary disease.

**Table 4:** Predictors of long-term mortality after isolated mitral valve replacement: univariate and multivariate cox proportional hazards regression analysis.

Variable	Univariate Analysis		Multivariate Analysis	
	HR (95% CI)	*p*	HR (95% CI)	*p*
Age (per year)	1.05 (1.04–1.07)	<0.001	1.04 (1.02–1.06)	<0.001
Female sex	1.48 (1.11–1.82)	<0.001	1.24 (1.06–1.55)	0.013
Presence of HT	1.10 (0.90–1.35)	0.364	—	—
Presence of DM	1.08 (0.84–1.39)	0.541	—	—
Presence of PH	1.75 (1.12–2.74)	0.013	1.43 (1.01–2.04)	0.045
Prior PCI	1.00 (0.68–1.48)	0.990	—	—
Presence of CPC	1.90 (1.10–3.30)	0.021	1.74 (1.08–2.81)	0.022
Presence of AF	1.01 (0.82–1.24)	0.913	—	—
Presence of CAD	0.91 (0.73–1.14)	0.432	—	—
Presence of COPD	1.05 (0.78–1.42)	0.751	—	—
Creatinine > 200 µmol/L	2.79 (1.90–4.10)	<0.001	2.26 (1.50–3.41)	<0.001
BMI (per kg/m^2^)	1.06 (1.03–1.09)	<0.001	1.04 (1.01–1.08)	0.014
CPB time (per min)	1.01 (0.99–1.02)	0.460	—	—
Cross-clamp time (per min)	1.01 (0.99–1.03)	0.410	—	—
LV function				
Good	Reference	—	Reference	—
Moderate	1.18 (0.91–1.54)	0.211	1.12 (0.84–1.49)	0.440
Poor	2.12 (1.48–3.03)	<0.001	1.87 (1.26–2.77)	0.002

Abbreviations: HR, hazard ratio; CI, confidence interval; BMI, body mass index; CPB, cardiopulmonary bypass; ACC, aortic cross-clamp; HT, hypertension; DM, diabetes mellitus; PH, pulmonary hypertension; CPC, critical preoperative condition; AF, atrial fibrillation; CAD, coronary artery disease; LV, left ventricular; PCI, percutaneous coronary intervention; COPD, chronic obstructive pulmonary disease.

## Data Availability

The data supporting the findings of this study are available from the corresponding author upon reasonable request. The data are not publicly available due to ethical and privacy restrictions related to patient confidentiality.
